# Role of human tissue kallikrein in gastrointestinal stromal tumour invasion

**DOI:** 10.1038/sj.bjc.6605906

**Published:** 2010-09-21

**Authors:** P Dominek, P Campagnolo, M H-Zadeh, N Kränkel, M Chilosi, J A Sharman, A Caporali, G Mangialardi, G Spinetti, C Emanueli, M Pignatelli, P Madeddu

**Affiliations:** 1 Experimental Cardiovascular Medicine, Bristol Heart Institute, University of Bristol, Level 7, Bristol Royal Infirmary, Upper Maudlin Street, Bristol, UK; 2 Clinical Science at South Bristol, University of Bristol, Level 7, Bristol Royal Infirmary, Upper Maudlin Street, Bristol, UK; 3 University of Verona, Verona, Italy; 4 Vantia Ltd, Southampton Science Park, Southampton, UK; 5 Multimedica IRCCS, Milan, Italy

**Keywords:** kallikrein, angiogenesis, invasion, GIST

## Abstract

**Background:**

Human tissue kallikrein (hK1) generates vasodilator kinins from kininogen and promotes angiogenesis by kinin-dependent and kinin-independent mechanisms. Here, we investigate the expression and functional relevance of hK1 in human gastrointestinal stromal tumour (GIST).

**Methods:**

Vascularisation and hK1 expression of GIST samples were assessed by immunohistochemistry. In two GIST cell lines, hK1 expression was assessed by PCR, and hK1 protein levels and activity were measured by ELISA and an amidolytic assay, respectively. The effect of hK1 silencing, inhibition or overexpression on GIST cell proliferation, migration and paracrine induction of angiogenesis was studied. Finally, local and systemic levels of hK1 were assessed in mice injected with GIST cells.

**Results:**

Human tissue kallikrein was detected in 19 out of 22 human GIST samples. Moreover, GIST cells express and secrete active hK1. Titration of hK1 demonstrated its involvement in GIST invasive behaviour, but not proliferation. Furthermore, hK1 released by GIST cells promoted endothelial cell migration and network formation through kinin-dependent mechanisms. Gastrointestinal stromal tumour implantation in nude mice resulted in local and systemic hK1 expression proportional to tumour dimension.

**Conclusions:**

Human tissue kallikrein is produced and released by GIST and participates in tumour invasion. Further studies are needed to validate hK1 as a diagnostic biomarker and therapeutic target in GIST.

Gastrointestinal stromal tumour (GIST) is one of the most common mesenchymal tumours of the gastrointestinal tract, the clinical features of which comprise acute or chronic bleeding, altered bowel function and intestinal obstruction or perforation, depending on the size and location of the tumour (Miettinen and Lasota, 2006). Approximately 85–90% of GISTs harbour activating mutations for the stem cell factor (SCF) receptor CD117 (c-Kit) or the alpha-type platelet-derived growth factor receptor (PDGFR*α*) (Heinrich *et al*, 2000; Hirota *et al*, 2000), which makes this tumour responsive to the tyrosine kinase inhibitor imatinib mesylate (Demetri *et al*, 2002). However, the mechanisms involved in the invasive capacity of GIST remain largely unknown.

The kallikrein (hK) family has been recognised to have fundamental roles in cancer and vascular biology (Bhoola *et al*, 2001; Borgono and Diamandis, 2004; Clements *et al*, 2004; Madeddu *et al*, 2007). Individual members of the hK family have in the past been identified as biomarkers for cancer, such as prostate cancer-specific marker hK3 (Welch and Albertsen, 2009). To date, no studies have investigated the involvement of hKs in the growth and development of GIST.

Human tissue kallikrein (hK1) has a crucial role in postischaemic neovascularisation (Emanueli *et al*, 2000, 2001, 2004; Yao *et al*, 2008; Stone *et al*, 2009). Furthermore, hK1 has been implicated in the growth and invasiveness of pancreatic carcinoma (Wolf *et al*, 2001), oesophageal carcinoma (Dlamini *et al*, 1999; Dlamini and Bhoola, 2005), gastric malignancy (Sawant *et al*, 2001) and lung cancer (Chee *et al*, 2008). Although the exact molecular mechanisms by which hK1 promotes tumour growth and invasion have not been determined so far, two principal actions of hK1 may have a role: (1) promoting tumour cell invasion of extracellular matrix by its protease activity, directly or through the activation of metalloproteinases (MMPs) (Tschesche *et al*, 1989; Desrivieres *et al*, 1993; Menashi *et al*, 1994; Leeb-Lundberg *et al*, 2005; Stone *et al*, 2009) and (2) activating kinin receptors, either directly (Biyashev *et al*, 2006) or through the generation of kinins (Madeddu *et al*, 2007). Autocrine activation of the kinin B2 receptor (B2R) on tumour cells may promote proliferation and motility, whereas paracrine action could induce endothelial cell (EC) proliferation and migration, thus increasing tumour vascularisation.

In this study, we aimed to investigate whether hK1 is expressed and released by GIST and participates in tumour growth and expansion.

## Materials and methods

### Immunohistochemistry

Formalin-fixed and paraffin-embedded GIST specimens were obtained from the Departments of Pathology of Bristol University (UK) and Verona University (Italy), with the approval of the local ethics committee. Xenograft tumour samples were obtained after excision from euthanised animals. Serial sections (3 *μ*m) were incubated overnight with monoclonal antibodies antihuman hK1 (1 : 100, R&D) and CD31 (1 : 40, DAKO) or Isolectin B4 (1 : 100, Sigma, St Louis, MO, USA) at 4 °C. Immunoreactivity was detected using the ABC method (Vector Labs, Burlingame, CA, USA) or by applying a suitable fluorescence-labelled secondary antibody. Normal mouse IgG (Santa Cruz, Santa Cruz, CA, USA) was used as a negative control.

Images were taken at × 200 and × 400 magnification using an Olympus light microscope (BX 40, Southall, UK). The stained area and intensity score were evaluated in the GNU Image Manipulation Program (GIMP) using colour selection and histogram function. HK1 staining intensity was recalculated to a 0–100% scale, where zero represents no staining and 100 represents the darkest (black) area. In CD31-stained samples, vessels were expressed as the average number of vessels per section.

### GIST cell line

The immortalised GIST882 and GIST48 cell lines were a kind gift from Dr J Fletcher (Brigham and Women's Hospital, Boston, MA, USA) (Tuveson *et al*, 2001). GIST882 was cultured in RPMI 1640 supplemented with 15% FBS, 2 mM L-glutamine, 100 IU ml^−1^ penicillin and 100 *μ*g ml^−1^ streptomycin (Cambrex, East Rutherford, NJ, USA). GIST48 was cultured in F10 (GIBCO) with 20% FBS, 2 mM L-glutamine, 100 IU ml^−1^ penicillin and 100 *μ*g ml^−1^ streptomycin, MITO serum extender and bovine pituitary extract (BD).

Human umbilical vein endothelial cells (HUVEC, Cambrex) were grown in EGM-2 containing 2% FBS (Lonza, Basel, Switzerland).

### Conditioned medium

Highly confluent GIST882 or GIST48 cells were incubated with serum-free medium for 24 h. After medium collection, cells were trypsinised and counted to establish total number.

### hK1 silencing

Three to five million GIST882 cells were transfected with 150 nM of small interfering RNA (siRNA) for hK1 or scrambled control using Amaxa electroporation protocol (Amaxa Biosystems, Gaithersburg, MD, USA). Effective silencing was verified by RT–PCR and ELISA (see below). On-target plus siRNA was purchased from Dharmacon (Lafayette, CO, USA).

### Infection with adenoviral vector carrying hK1

GIST882 cells were incubated with 50 moi (multiplicity of infection) of *Ad.hK1* or *Ad.Null* overnight. Experiments were performed 24 h later. Successful infection was verified by RT–PCR (see below). Adenoviruses were prepared as previously described (Stone *et al*, 2009).

### Measurement of hK1 levels and activity

Immunoreactive hK1 in cell culture supernatants and blood was measured by ELISA as previously reported (Wang *et al*, 1995; Porcu *et al*, 2002, 2004) and hK1 activity was assayed using the chromogenic substrate S-2266, following the manufacturer's instructions (Chromogenix, Milano, Italy) and as previously described (Madeddu *et al*, 1993).

### Expression analysis

RNA was obtained using an RNeasy Minikit (Qiagen, Crawley, UK) and reverse transcribed with MMLV (Invitrogen, Paisley, UK). Real-time PCR was carried out with PCR Mastermix 2x (Promega, Madison, WC, USA).

Primers were designed for hK1: Forward 5′-GGGTCGCCACAACTTGTTTG-3′ and Reverse 5′-GCTGTAGTCCTCGTCTGCTT-3′ B1R: Forward 5′-CTTCCCTCAAAATGCTACGGC-3′ and Reverse 5′-TCTGCCACGTTCAGTTGCC-3′ B2R: Forward 5′-GTCTGTTCGTGAGGACTCCG-3′ and Reverse 5′-CTGGGCAAAGGTCCCGTTAAG-3′ ACE: Forward 5′-AACGAAACCCACTTTGATGC-3′ and Reverse 5′-TCAGCCTCATCAGTCACCAG-3′ HMK: Forward 5′-TGGGGCCATGAAAAACAAAG-3′ and Reverse 5′-CTTGGCTAGGGAAGGGATGG-3′ LMK: Forward 5′-CCAGCATCTGAGAGGGAGGT-3′ and Reverse 5′-GCAGAATGGGTAGGGCTGAA-3′.

### BrdU cell proliferation assay

Gastrointestinal stromal tumour cells were seeded in 96-well plates (5000 cells per well) and incubated with BrdU for 24 h in normal medium or in the presence of 0.5, 0.05 or 0.005 *μ*M of specific hK1 inhibitors VA999154 or VA999024, kindly provided by Vantia Ltd. (Chilworth, UK) BrdU incorporation was determined following the manufacturer's protocol (Roche, Melwin, UK).

### GIST invasion assay

The invasion capacity of GIST882 and GIST48 cells was measured by seeding 5 × 10^4^ cells on top of an 8 *μ*m filter coated with Matrigel (BD, 1 : 100). Medium containing 5% FBS was used as a stimulus. hK1 inhibitors VA999154 and VA999024 were added at a concentration of 0.5 *μ*M as described above. After 24 h, the filters were mounted with DAPI to recognise the nuclei of migrated cells. Cell number was calculated by averaging the counts of five microscopic fields (photographed at × 20).

### Endothelial cell migration assay

The migratory response of ECs to GIST was assessed in a transwell array (Corning, Corning, NY, USA). Briefly, 3 × 10^5^ GIST882 cells were plated on the bottom chamber of the migration system. Human umbilical vein ECs (HUVECs) were preincubated with B_1_R or B_2_R antagonists (Lys-des-Arg^9^Leu^8^-BK, LdL-BK or Icatibant, IC, respectively, 2 × 10^−7^M) or PBS (vehicle), plated on top of the insert (50 000 cell per insert) and then left to migrate overnight in the presence or absence of B_1_R or B_2_R antagonists. Migrated HUVECs were counted as described for the invasion assay and the percentage of migrated cells was calculated on the number of plated cells.

### Matrigel angiogenesis assay

HUVECs pretreated for 30 min with the hK1 inhibitor kallistatin (1 *μ*M, R&D) (Wolf *et al*, 1999), the serine protease inhibitor Aprotinin (50 U ml^−1^, Bayer, FRG) or vehicle were plated on growth factor-reduced Matrigel (BD Biosciences, Erembodegem, Belgium) in the presence of GIST882-conditioned medium or unconditioned medium (as a control). Total tube length and average tube thickness were measured on photographs captured at 24 h and analysed using ImagePro Plus software (Media Cybernetics, Bethesda, MD, USA).

### FACS analysis

Cells were incubated with rabbit polyclonal antibodies for B_1_R and B_2_R (1 : 100, Sigma), followed by FITC-labelled antirabbit secondary antibodies (Sigma) and analysed in a FACScalibur (BD Biosciences) flow cytometer. Controls were stained with isotype control.

### Xenograft model

The experiments involving mice were conducted in accordance with the Guide for the Care and Use of Laboratory Animals prepared by the Institute of Laboratory Animal Resources and with previous approval of the UK Home Office and the University of Bristol ethics committee.

GIST882 cells were implanted under the dorsal skin of BALB/c nude mice in three doses (3 × 10^6^-3M, 5 × 10^6^-5M and 8  × 10^6^-8M cells or vehicle; four mice per dosage). Tumour growth was monitored biweekly. All mice were euthanised with an excess of anaesthesia when the tumour size of the highest-dose group reached 16 mm^3^. Before being killed, blood was collected by cardiac puncture and tumour tissue was collected for further analysis.

### Statistical analysis

Continuous variables were compared by one-way ANOVA or Student's *t*-test as appropriate. Relationships between variables were determined by the Pearson correlation coefficient. Continuous data are expressed as mean±s.e.m. A *P* value <0.05 was considered statistically significant. Analyses were performed with GraphPad Prism 5.0 (Graphpad software, La Jolla, CA, USA).

## Results

### High circulating levels of hK1 in a GIST patient

**Figure 1:**
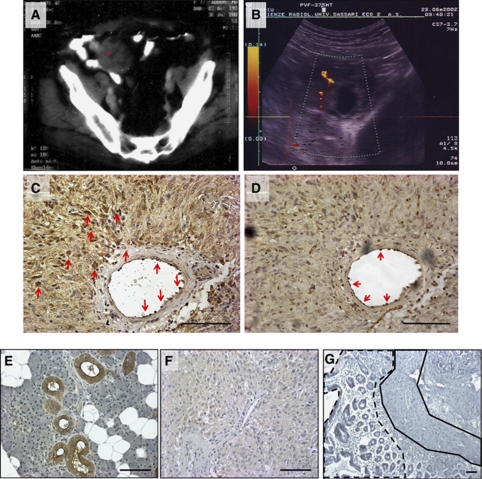
Clinical case. Representative CT scan images showing the presence of a tumour mass, highlighted by an asterisk, in the abdominal cavity (**A**). The mass appears irregularly perfused, as shown by Doppler imaging (**B**). Immunohistochemistry analysis confirmed typical GIST characteristics and expression of hK1 (red arrows) in tumour cells (**C**) and vasculature (**D**, stained with von Willebrand). Specificity of the hK1 staining was confirmed on sections of salivary glands (**E**, positive control) and GIST incubated with mouse IgG (**F**, negative control). Biopsies derived from normal gastrointestinal tract reveal no expression of hK1 in either the epithelial layer (dashed line) or in the muscular area (continuous line) (**G**). Scale bar: 100 *μ*m.

We previously reported that hK1 levels are remarkably elevated in peripheral blood of patients with critical carotid artery obstruction and normalised after endarterectomy (Porcu *et al*, 2004). Only one subject of this series showed persistently high hK1 levels before and after revascularisation (1456 and 1681 pg ml^−1^, respectively). The patient was referred to us 8 months after endarterectomy because of the appearance of constipation. Computerised tomography scanning documented a solid mass (7 × 6 cm diameters) infiltrating the ileum and reaching the abdominal wall (Figure 1A). At power Doppler, the mass appeared irregularly perfused (Figure 1B). Surgical resection resulted in a remarkable reduction of circulating hK1 (368 pg ml^−1^, 2 months after surgery). Histological examination of the mass revealed the characteristics of mixed spindle and epithelioid cell GIST, positive for c-Kit and negative for S-100 protein, glial fibrillary acidic protein (GFAP), desmin and CD34 (not shown). Vascular cells (EC) and GIST cells were positive for hK1 (red arrows, Figure 1C). The vascular endothelium was identified in consecutive sections by positive staining for von Willebrand factor (Figure 1D). Specificity of the reaction was confirmed by parallel staining of hK1-producing salivary glands and GIST specimens (Figure 1E and F). Normal gastrointestinal tissue was negative, thus confirming the aberrant expression of hK1 by GIST (Figure 1G).

### Retrospective analysis of hK1 expression in GIST

**Figure 2:**
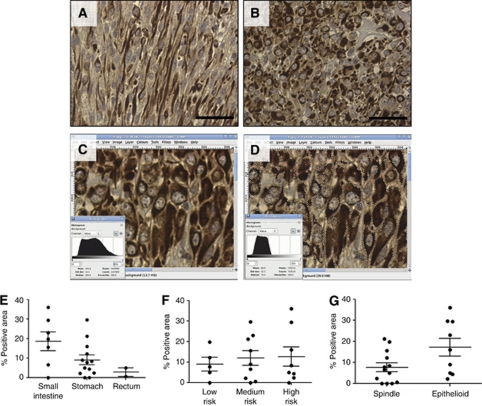
Immunohistochemical localisation of hK1 in human GIST samples. Microphotographs show strong cytoplasmatic staining for hK1 in the tumour tissue in both spindle-shaped (**A**) and epithelioid cells (**B**). Scale bar: 100 *μ*m. For each section, the area positive for hK1 was selected and the ratio between the pixels of the selected area and total number of pixels was calculated (in the example, 733 116/1 447 680 × 100=56%). Histograms show the analysis of corresponding images. Average colour intensity in the selection was considered as staining intensity (median=70 in the bottom right histogram **C** and **D**). Scatterplot graphs showing the relationship between the calculated expression of hK1 and GIST localisation (**E**), risk (**F**) and cell type (**G**). Each dot represents a GIST case.

We verified the expression of hK1 in a series of 22 GIST cases. Patient characteristics are summarised in Table 1, which also reports tumour localisation and risk of aggressive behaviour as proposed by Fletcher *et al* (Fletcher *et al*, 2002). Immunohistochemistry revealed that 19 of 22 GIST cases were positive for hK1 with variable intensity and percentage of positive tissue (representative microphotographs in Figure 2A and B).

Quantitative assessment of hK1 expression was performed by measuring (1) the section area positive for hK1 in relation to the total section area and (2) the intensity of the staining (Figure 2C and D). As shown in Figure 2E, ANOVA detected a difference in hK1 expression with regard to the tumour location (*P*=0.02), with largest positive areas in small intestine samples (18.6±4.7% of total section), followed by the stomach (9.1±2.4%) and rectum (2.9±2.2%). In contrast, hK1 expression was independent of the risk index or cellular type (Figure 2F and G). Furthermore, no correlation was found with the tumour vascular density, adjusting for age, sex, tumour cellular type, location and risk index.

### GIST cell lines express hK1

**Figure 3:**
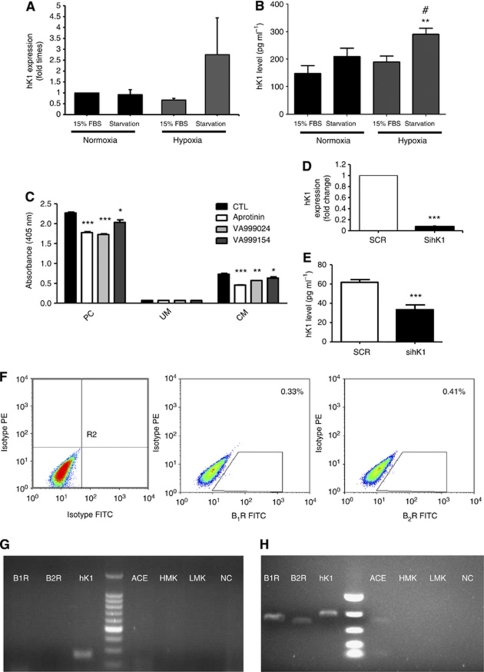
Gastrointestinal stromal tumour cell expression of kallikrein system components. Bar graphs show the effect of hypoxia and serum deprivation (starvation) on hK1 mRNA (**A**) and protein secretion into culture medium (**B**). HK1 mRNA was normalised for 18S and expressed as fold change *vs* 15% FBS. Values are mean±s.e.m. of *n*=6 replicates. ^**^P<0.01 *vs* normoxia+15%FBS and*P*<0.05 *vs* hypoxia+15%FBS. (**C**) GIST882 cells release enzymatically active hK1 in the conditioned medium (CM). Urine was used as a positive control (PC). Unconditioned medium (UM) was included as a negative control. Values are mean±s.e.m. of two measurements. ^*^P<0.05, ^**^P<0.01 ^***^P<0.001 *vs* non-inhibited reaction (CTL). Effective hK1 silencing (sihK1) was verified by RT–PCR (**D**) and ELISA on conditioned media (**E**). Human tissue kallikrein mRNA was normalised for 18S and expressed as fold change *vs* SCR. Values are representative of three independent experiments. ^***^P<0.001 *vs* respective control. SCR=scramble. Flow cytometry analyses show that GIST882 cells do not express B_1_R and B_2_R, angiotensin-converting enzyme (ACE) or low (LMK) and high-molecular weight kininogen (HMK) (**F** and **G**). GIST48 RT–PCR analysis shows expression of both kinin receptors, hK1 and ACE (**H**). Negative control (NC) is the reaction run without cDNA.

Two different GIST cell lines were analysed for hK1 expression using RT–PCR and ELISA. Both Imatinib-sensitive GIST882 cells and Imatinib-resistant GIST48 express hK1 mRNA and protein. In particular, GIST882 cells release 30–400 pg ml^−1^ and GIST48 cells about 40 pg ml^−1^ of immunoreactive hK1 into the culture medium. Furthermore, the exposure of GIST882 cells to hypoxia and serum deprivation increased hK1 mRNA levels (Figure 3A) and immunoreactive hK1 content in the culture medium (Figure 3B). To verify that secreted hK1 is enzymatically active, GIST882 supernatants were assayed using a colorimetric assay for hK1 in the presence or absence of the serine protease inhibitor aprotinin and specific inhibitors VA999154 and VA999024. Results showed the presence of inhibitable enzymatic activity in GIST882 supernatants (Figure 3C). Lower levels of hK1 expression detected in GIST48 supernatants were not sufficient for the enzymatic activity quantification (not shown). Levels of hK1 mRNA and protein could be efficiently inhibited using siRNA (Figure 3D and E).

### Effect of hK1 on GIST882 cell function

Human tissue kallikrein might be implicated in GIST growth and invasiveness through an autocrine mechanism mediated by kinin receptors, as well as through promotion of extracellular matrix degradation.

Flow cytometry (Figure 3F) and RT–PCR (Figure 3G) demonstrated the absence of B_1_R and B_2_R protein and mRNA in GIST882 cells. Furthermore, GIST882 cells do not express kininogens or the kinin-degrading enzyme angiotensin-converting enzyme (ACE). Conversely, GIST48 showed expression of both receptors and ACE (Figure 3H). These data indicate that, although hK1 is the only component of the kallikrein–kinin system expressed by GIST882 cells, therefore excluding kinin receptor-mediated autocrine mechanisms in these tumoural cells, GIST48 might respond directly to hK1.

The effect of changes in hK1 expression on GIST882 cell functional properties was then studied using siRNA-mediated silencing, inhibition by VA999154 and VA999024, or adenovirus-mediated *hK1* gene transfer. Efficient transduction after gene transfer was confirmed by ELISA showing a 3 × 10^3^-fold increase in hK1 levels compared with *Ad.Null* (data not shown).

**Figure 4:**
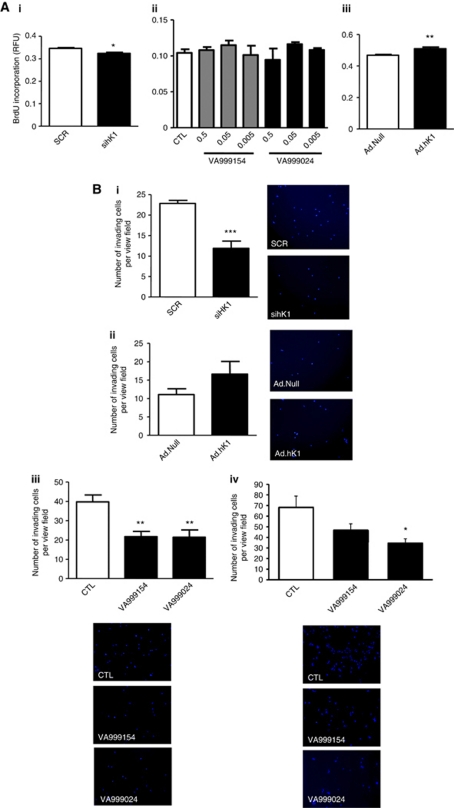
Human tissue kallikrein is implicated in the invasive capacity of GIST cells. Gene silencing by sihK1 or forced expression of hK1 by Ad.hK1 produced mild reciprocal effects on GIST882 cell proliferation, whereas pharmacological inhibition was ineffective (**Ai**–**Aiii**). Invasion activity of GIST882 cells was strongly reduced by sihK1 compared with scramble (SCR), conversely, infection with *Ad.hK1* enhanced GIST882 invasion as compared with *Ad.Null* (**Bi** and **Bii**). Pharmacological inhibition of hK1 (VA999154 and VA999024) significantly reduced GIST882 and GIST48 invasion capacity (**Biii** and **Biv**) Values are mean±s.e.m. of *n*=3 experiments, each performed in quadruplicate. ^*^P<0.05, ^**^P<0.01 and ^***^P<0.001 *vs* respective control (empty bar).

A very small effect of hK1 silencing on GIST882 proliferation was detected (Figure 4Ai), but not confirmed, by pharmacological inhibition (Figure 4Aii). Similarly, a very small, but significant increase in GIST882 proliferation following hK1 transduction was observed (Figure 4Aiii). Pharmacological inhibition of hK1 did not exert significant effects on GIST48 proliferation (data not shown).

Silencing of hK1 significantly decreased, whereas hK1 overexpression tended to increase GIST882 cell invasive capacity (Figure 4Bi and Bii). Pharmacological inhibition of hK1 reduced both GIST882 and GIST48 invasiveness, suggesting that hK1 might be involved in GIST dissemination (Figure 4Biii and Biv).

### Endothelial cell migration towards GIST is mediated by kinin receptors

Previous studies highlighted the role of endogenous and transgenic hK1 in the promotion of angiogenesis (Emanueli *et al*, 2000, 2001; Stone *et al*, 2009). Here, we assessed whether GIST-released hK1 may exert a proangiogenic action on HUVEC, thus supporting an attractive effect on host vessels.

**Figure 5:**
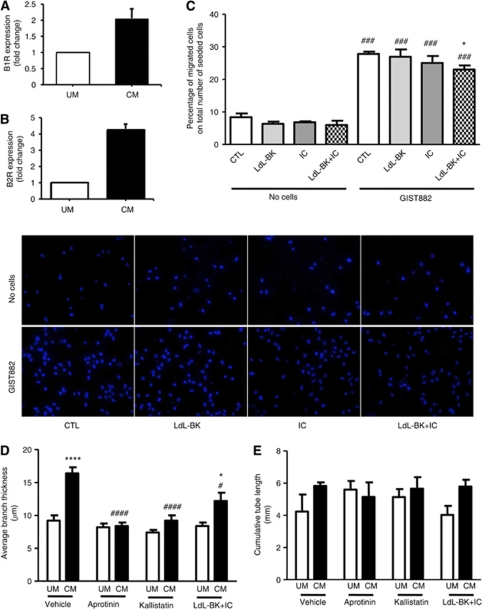
GIST882 cells stimulate endothelial cell migration and network formation capacity by a mechanism involving hK1 and kinin receptors. Bar graphs showing the expression levels of B_1_R (**A**) and B_2_R (**B**) in HUVEC incubated with GIST882-conditioned medium (CM) or unconditioned medium (UM). Values were normalised for 18S and expressed as fold change *vs* UM. Bar graph shows the effect of GIST882 cells on HUVEC migration capacity. The presence of GIST in the bottom chamber enhanced HUVEC migration. Addition of B_1_R (Lys-des-Arg^9^Leu^8^-BK, LdL-BK) and B_2_R (Icatibant, IC) antagonists did not have any effect on spontaneous migration as compared with the control (CTL), but reduced GIST-dependent migration (**C**). Bar graph shows the effect of GIST882-conditioned medium (CM) as compared with unconditioned medium (UM) on tube width (**D**) and tube length (**E**) in networks formed by HUVEC on Matrigel, in the presence or absence of aprotinin, kallistatin of kinin receptor antagonists. Values are mean±s.e.m. of six measures. ^###^*P*<0.001 *vs* no cells or vehicle; ^*^P<0.05 and ^****^P<0.001 *vs* vehicle or UM.

Exposure of HUVECs to GIST882-conditioned medium increased B_2_R mRNA expression by four-fold, and B_1_R by two-fold (Figure 5A and B). In a transwell migration assay, GIST882 cells strongly attracted HUVECs. Addition of B_1_R or B_2_R antagonists had only a mild effect on HUVEC migration, whereas a 25% inhibition was obtained with the combination of both compounds (Figure 5C). These data suggest that B_1_R and B_2_R are involved in GIST-induced HUVEC migration.

We then verified whether GIST882-conditioned medium has a stimulatory action on HUVEC network formation capacity in the Matrigel assay. We found that GIST882-conditioned medium stimulated the formation of wider branches, with this effect being inhibited by aprotinin, the hK1 inhibitor kallistatin and, to a minor extent, the combination of B_1_R and B_2_R antagonists (Figure 5D). In contrast, GIST882-conditioned medium did not influence the cumulative length of branches (Figure 5E). These results indicate that hK1 secreted by GIST contributes to endothelial migration and promotes vascular stabilisation.

### Local and systemic expression of hK1 in mice with xenogeneic GIST graft

Finally, we tested whether xenogeneic GIST882 cell grafts in nude mice reproduce the phenotype observed in GIST-bearing patients, for example, local expression of hK1 and high hK1 circulating levels.

**Figure 6:**
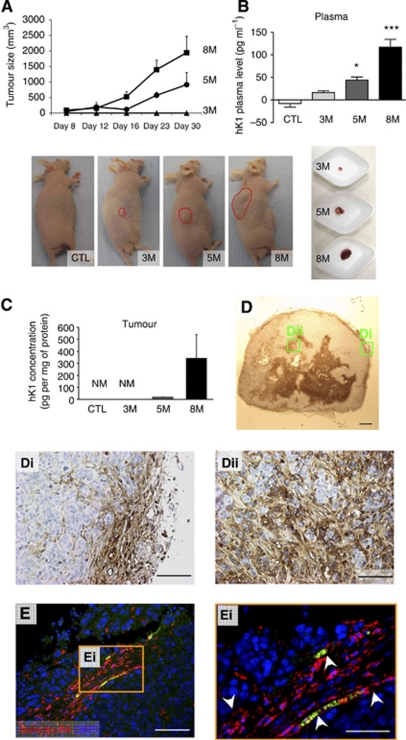
Expression of hK1 by GIST xenografts. Line graph showing the growth of GIST xenografts implanted in nude mice (**A**). Three cell dosages were injected (3 × 10^6^ (3M), 5 × 10^6^ (5M) and 8 × 10^6^ (8M) cells or vehicle; four mice per dosage). Pictures show the appearance of tumour masses. Although no apparent growth or small nodules were observed with the lower dosage, higher dosages produced increasingly large masses. Bar graphs show the levels of immunoreactive hK1 in peripheral blood (**B**) and tumour nodules (**C**). NM=not measurable. Microphotographs show hK1-positive cells in tumour nodules (**D**). Microphotograph shows host-derived endothelial cells stained by Isolectin B4 (red, **E**) at the periphery of the tumour forming vascular structures (**Ei**, white arrowheads). Green staining is due to red blood cell autofluorescence. ^*^P<0.05 and ^***^P<0.001 *vs* vehicle (empty bar). Scale bar: 500 *μ*m (**D**), 100 *μ*m (**Di**–**ii** and **E**) and 50 *μ*m (**Ei**).

To this aim, three escalating GIST882 cell dosages (3 × 10^6^, 5 × 10^6^ and 8 × 10^6^) or vehicle was implanted under the skin of BALB/c nude mice. Tumour growth was directly proportional to the number of injected cells, with the lowest cell dosage (3 × 10^6^) barely forming identifiable nodules (Figure 6A). Similarly, circulating levels of hK1 increased in tumour-bearing mice in proportion to tumour size, being detectable even in the smallest dosage of GIST882 cells (Figure 6B). Tumours were explanted from mice given 5 × 10^6^ or 8 × 10^6^ GIST882 cells and subjected to verification of hK1 expression by ELISA and immunohistochemistry. Enzyme-linked immunosorbent assay revealed high levels of hK1 in the examined tumours (Figure 6C). Immunohistochemistry confirmed the cytoplasmatic staining for hK1 as observed in human GIST sections (Figure 6D). In particular, hK1 was expressed in the central core and the peripheral part of the tumour. Analysis of tumour vasculature identified host vessels invading the periphery of the xenograft (Figure 6E).

## Discussion

A significant number of adenocarcinomas express hKs. These tumours likely take advantage of hKs enzymatic properties to influence growth and metastasis (Borgono and Diamandis, 2004). Despite its early discovery, association of hK1 with cancer was late in comparison with other members of the hK family. A previous study showed the expression and localisation of hK1 in pancreatic adenocarcinoma of ductal origin and in a breast cancer cell line (Wolf *et al*, 2001). To the best of our knowledge, this is the first documentation that GISTs, the most common mesenchymal tumour of the gastrointestinal tract, express and release hK1 into the surrounding environment and circulation. The reason for this aberrant expression remains unknown. HK1 expression is responsive to oestrogens (Madeddu *et al*, 1991); however, we could not find a gender-related difference in hK1 levels in the GISTs of our series. Furthermore, hK1 levels were not associated with the cellular type and indexes of aggressiveness. An association was instead found with tumour location, with the highest values in GISTs of the small intestine. Our *in vitro* data show that expression levels in the GIST cell line are increased by hypoxia/starvation, pointing to the possibility that areas of the tumour far away from the vasculature could be stimulated to produce larger amounts of hK1. This is, at least in part, in line with the prevalent localization of hK1 in the central avascular core of GIST882 xenografts.

*In vitro* studies on two different cell lines derived from Imatinib-resistant or Imatinib-sensitive tumours showed the common expression of hK1. Cell biology results suggest two distinct molecular mechanisms by which hK1 could be implicated in GIST growth: (1) invasion through degradation of the ECM and (2) induction/stabilisation of host-derived tumour vasculature. A direct action of hK1 as an ECM-degrading enzyme could be complemented by the ability of hK1 to cleave and activate other proteases, such as pro-MMPs (Menashi *et al*, 1994). The proinvasive action was verified by a gene titration approach, using siRNA to decrease native hK1 expression and *Ad.hK1* to force hK1 production. Silencing resulted in inhibition of GIST invasive capacity and overexpression enhanced it. The effect of silencing was further confirmed using inhibitors of hK1 activity. It is therefore likely that changes in hK1 production, release and clearance by endogenous inhibitors may confer a different invasive profile to the tumour.

Coculture experiments showed that GIST882 cells exert an attractive action on HUVEC, which is at least in part due to activation of kinin receptors, as verified by the use of receptor antagonists. Furthermore, GIST882-conditioned medium stimulates HUVEC to form more robust branches than those observed using unconditioned medium. The fact that kallistatin, which rapidly binds hK1 and inhibits its activity *in vitro* (Zhou *et al*, 1992), blocks the strengthening action of GIST882 cells on HUVEC networks argues in favour of hK1 as a promoter of cancer angiogenesis. Kallistatin itself was previously identified as an inhibitor of angiogenesis in gastric carcinomas (Zhu *et al*, 2007). In the retrospective analysis of human GISTs, we could not find any correlation between hK1 expression and vascular density, suggesting that other mechanisms may overwhelm or confound the proangiogenic effect of hK1 *in vivo*. On the other side, although HUVECs are widely used as models for tumoural angiogenesis, different mechanisms might drive cancer endothelial cells.

In conclusion, results of our study show for the first time that hK1 is implicated in GIST invasion and angiogenesis. GIST882 xenografts express and release hK1 into the circulation, a result that calls for further validation of hK1 as a potential diagnostic biomarker. On a therapeutic standpoint, hK1 inhibitors showed promising results in models of breast cancer invasion (Wolf *et al*, 2001). Combination therapies incorporating hK1 inhibition/silencing as adjuvant to imatinib mesylate therapy may be useful for the treatment of GIST.

## Figures and Tables

**Table 1:** Patients and tumours characteristics

Number of cases	22
Average age (years)	60±2
	
*Sex*	
Male	11
Female	11
	
*Location*	
Stomach	13
Small bowel	7
Rectum	2
	
*Risk level*	
Low	5
Intermediate	9
High	8
	
*Main cell type*	
Spindle	13
Epithelioid	8
Mixed	1
